# Morpho-physiological traits, biochemical response and phytoextraction potential of short-term copper stress on kenaf (*Hibiscus cannabinus* L.) seedlings

**DOI:** 10.7717/peerj.8321

**Published:** 2020-01-30

**Authors:** Muhammad Hamzah Saleem, Shah Fahad, Muzammal Rehman, Shah Saud, Yousaf Jamal, Sajjad Khan, Lijun Liu

**Affiliations:** 1 MOA Key Laboratory of Crop Ecophysiology and Farming System in the Middle Reaches of the Yangtze River, College of Plant Science and Technology, Huazhong Agriculture University, Wuhan, Hubei, China; 2 Department of Agriculture, University of Swabi, Swabi, Khyber Pakhtunkhwa, Pakistan; 3 College of Horticulture, Northeast Agricultural University, Harbin, Heilongjiang, China; 4 Crops Sciences Institute, National Agricultural Research Center (NARC), Islamabad, Pakistan; 5 School of Agriculture, Yunnan University, Kunming, China

**Keywords:** Translocation factor, Kenaf, Copper, Oxidative stress, Antioxidants, Gaseous exchange attributes, Bioaccumulation factor

## Abstract

Kenaf (*Hibiscus cannabinus* L.) is a fibrous crop, grown in tropical climate having huge biomass and can be a good candidate for the phytoremediation of different heavy metals. Consequently, the present study was conducted to explore morpho-physiological traits, photosynthetic pigments, gaseous exchange attributes, antioxidative response and phytoextraction of copper (Cu) in *H*. *cannabinus* grown under different levels of Cu i.e. 0 (control), 60, 120 and 180 µmol L^−1^ in Hoagland nutrient solution (pH 6.2). The results from the present study revealed that Cu toxicity reduced plant height, plant diameter, plant fresh weight, plant dry weight, photosynthetic pigments and gaseous exchange attributes compared to control. Moreover, excess Cu in the nutrient solution ameliorates contents of malondialdehyde (MDA), hydrogen peroxide (H_2_O_2_) and electrolyte leakage (EL) which showed that Cu induced oxidative damage in the roots and leaves of *H*. *cannabinus*. The oxidative stress which was induced by a high concentration of Cu in the nutrient solution is overcome by enzymatic activities of antioxidants which increased with the increase in Cu concentration, i.e. 60 and 120 µmol L^−1^, while the addition of Cu (180 µmol L^−1^) caused a reduction in the activities of superoxidase dismutase (SOD), peroxidase (POD), catalase (CAT) and ascorbate peroxidase (APX) in the roots and leaves of *H*. *cannabinus*. The results also demonstrated that an increase in Cu concentration in the nutrient solution causes an increase in Cu accumulation through roots, leaves and stems of *H*. *cannabinus*, although the highest Cu concentration was accumulated in roots while only a little transported to the above ground parts (leaves and stems) of the plants. All the values of bioaccumulation factor (BAF) and translocation factor (TF) were less than 1, which also indicated that a small quantity of Cu concentration is transported to the aboveground part of the plants. These findings suggested that phytotoxicity of Cu affected plant growth and biomass and increased ROS production while accumulation of Cu in different parts of plant proved that *H*. *cannabinus* is an ideal specie for phytoremediation of Cu when grown under Cu contaminated sites.

## Introduction

Heavy metals are considered as toxic metal pollutants in the environment, and even at low concentrations may cause many toxic impacts on plants (Gonzalez et al., 2010; Rehman et al., 2019b; Zhang et al., 2014). Heavy metals such as copper (Cu), nickel (Ni), cadmium (Cd), lead (Pb) and Zinc (Zn) are the major environmental pollutants, especially in areas with highly anthropogenic activities. Distribution of heavy metals in the soil is impacted by climatic and pedological factors such as industrial wastes and effluents, mining, smelting, and the use of many important fertilizers such as pesticides and fungicides (Li et al., 2009; Rehman et al., 2019a; Yu et al., 2016). Among different toxic metals, Cu is an important heavy metal and its toxicity is a problem with increasing significance for ecological and evolutionary reasons. However, Cu is an essential micronutrient for the plants, and is required in minute quantities for many biological and physiological processes in plants (Chen et al., 2015b; Husak, 2015; Li et al., 2018; Rehman et al., 2019a). Cu is an important part of photosynthesis, respiration, glucose, proteins and cell wall metabolism while excess Cu in the plants can cause alteration in DNA, cell membrane integrity, enzyme activity which ultimately affect crop yield and plant productivity (Kohli et al., 2018; Lu et al., 2017; Rehman et al., 2019c; Rizwan et al., 2017). In China, more than 16% of agricultural land is contaminated by different heavy metals and about 2.1% of the land is contaminated by Cu (Chen et al., 2015b). The main reasons behind this is use of Cu-made agrochemicals, pesticides, fungicides, bactericides and nematicides which enhances crop yield and productivity and control pests but that are major sources of high concentration of Cu in agricultural soil (Husak, 2015; Rehman et al., 2019b). The phytotoxicity of Cu in reducing the growth and biomass of the plants has been reported in many previous studies (Celis-Plá et al., 2018; Chen et al., 2015a; Chen et al., 2015b; Gao et al., 2017; Liu et al., 2018; Rehman et al., 2019c; Zaheer et al., 2015). Toxic concentration of Cu in soil can cause nutrient imbalance by binding with organic matter, clay minerals, and hydrated oxides of iron (Fe), aluminum (Al), and manganese (Mn), which affects the plant productivity. Moreover, Cu in excess causes generation of reactive oxygen species (ROS) in the plant’s tissues such as superoxide radical (O^−2^), hydrogen peroxide (H_2_O_2_), singlet oxygen (^1^O_2_), which causes injurious effects on plant metabolism and cellular structure (Chen et al., 2015b; Gonzalez et al., 2010; Ibrahim, Chee Kong & Mohd Zain, 2017). High contents of malondialdehyde (MDA) causes membrane destabilization of cellular organelles and indicating the prevalence of oxidative stress (Husak, 2015; Kohli et al., 2018). The antioxidants such as superoxide dismutase (SOD), peroxidase (POD), catalase (CAT) and ascorbate peroxidase (APX) comes into play to reduce metal toxicity by scavenging of ROS. Cu in excess induce oxidative damage in the plant tissues reported by many researchers (Chen et al., 2015b; Ibrahim, Chee Kong & Mohd Zain, 2017; Kosar, Akram & Ashraf, 2015; Rehman et al., 2019c) and antioxidants like SOD, POD, CAT and APX withstands when plant undergoes stress conditions (Kamran et al., 2019; Rehman et al., 2019a).

Recently, phytoextraction, the use of green plant for the remediation of toxic heavy metals, has become increasingly acceptable due to cheap, eco-friendly, scientifically acceptable and extensively applicable technique (Chen et al., 2015b; Lajayer et al., 2019; Lu et al., 2017). Phytoextraction is best suitable for those plants whom having huge biomass, quick growth period and move toxic pollutants from the soil to harvestable parts (leaf and stem) of the plants. However, the efficiency of plants for accumulating these toxic pollutants from the soil and transport them to their aboveground parts depends upon the plant species, growth conditions and soil type (Ali & Chaudhury, 2016; Lu et al., 2017). According to Muszynska & Hanus-Fajerska (2015), more than 500 plant species has been used as hyperaccumulators for different heavy metals and able to accumulate high concentration of heavy metals in their plant tissues without any noxious effect. Previously, many different plant species used as hyperaccumulator for different heavy metals such as Cu, Zn, Pb and Cr (Celis-Plá et al., 2018; Chen et al., 2015b; Lajayer et al., 2019; Lu et al., 2017) while many studies have been conducted on different species of *Hibiscus* (Li et al., 2013; Nasrollahzadeh, Issaabadi & Sajadi, 2018). Kenaf (*Hibiscus cannabinus* L.) belongs to the family Malvaceae and is an herbaceous, annual fibrous crop which has been used as hyperaccumulator for different heavy metals due to its huge biomass (Ding et al., 2016; Ververis et al., 2016; Wuana & Mbasugh, 2013). Recent works of research and development have demonstrated the suitability of *H*. *cannabinus* for use in building materials, adsorbents, textiles and fibers for new and recycled plastics (Li et al., 2013; Othmana, Akil & Kimc, 2008). Furthermore, its fibers were also recommended as reinforcement fiber for high-performance biodegradable polymer composites. In addition, *H*. *cannabinus* is a potentially suitable species for phytoremediation in the tropics, with a combined purpose of biomass production to improve the ecological and economic value of degraded areas (Ho, Ang & Lee, 2008; Ververis et al., 2016). Moreover, among different species of *Hibiscus*, *H*. *cannabinus* is considered to be a more tolerant species to the metal contaminated soil as it has a deep rooting system, quick growth rate and specific biological and biochemical activities (Li et al., 2013; Santos et al., 2010).

Many investigations have been done about the potential of various plant species such as *Corchorus Capsularis*, *Boehmeria nivea* and *Triticum aestivum* (Gang, Vyas & Vyas, 2013; Rehman et al., 2019c; Uddin et al., 2016) for phytoremediation of toxic heavy metals but very few studies have explored the potential of *H*. *cannabinus* to remediate Cu-polluted soils. According to best of our knowledge, this study is among the few studies which focus on the metal tolerance and accumulation among fibrous crop in order to investigate their suitability for metal-contaminated sites. The results from this study will add to our knowledge about (i) the morpho-physiological responses of *H*. *cannabinus* to Cu, (ii) the Cu induced oxidative responses of *H*. *cannabinus* and (iii) Cu accumulation potential of *H*. *cannabinus* grown under various levels of Cu-polluted soil.

## Material and Methods

### Growth conditions and treatments

**Figure 1:**
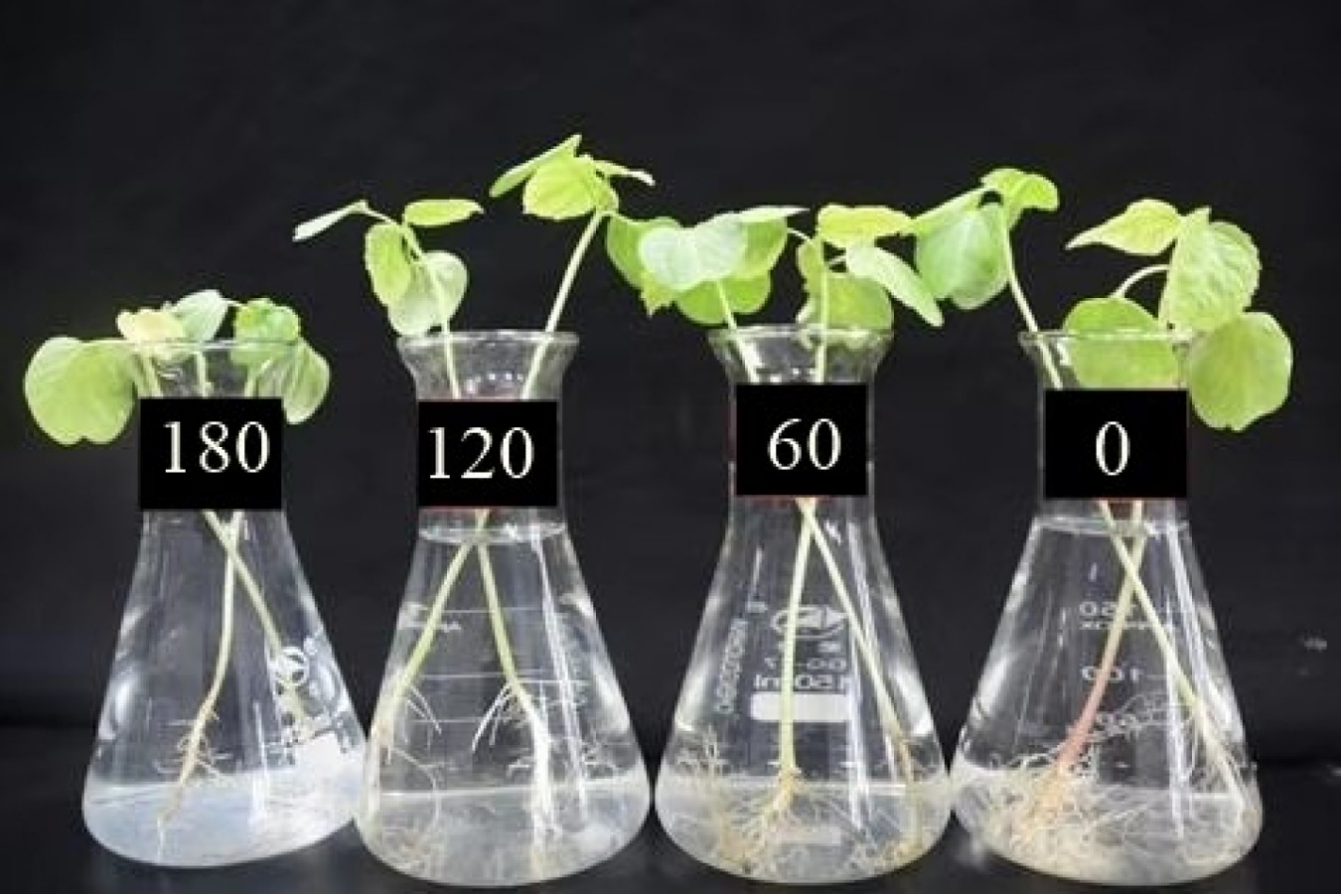
Effect of different levels of Cu concentration (0, 60, 120 and 180 µmol L^−1^) on *H. cannabinus* seedlings. Levels of Cu concentration (0, 60, 120 and 180 µmol L^−1^).

Mature seeds of *H*. *cannabinus* (HC-95) were thoroughly washed with 0.1% mercuric chloride (HgCl_2_) solution than rinsed with distilled water (Thounaojam et al., 2012) and sow in experimental stations of Huazhong Agricultural University Wuhan, China (114.20′ E, 30.28′ N) on March 1st 2019. After two weeks uniform sized rhizomes were collected and transferred into flask (150 ml) containing nutrient Hoagland solution (pH 6.2) and placed in growth chamber where they received 12 h natural with humidity 70% at 25 °C (Philips 20W TLD, China). The nutrient solutions were artificially spiked with Cu at various levels i.e., 0 (control), 60, 120 and 180 µmol L^−1^ using CuSO_4_. 5H_2_0 (99% purity) (Fig. 1). The experiment was arranged in a complete randomized design (CRD) with six replications and two plants were kept in each flask. Cu spiked nutrient solutions were vigorously aerated and replaced the nutrient solution three times in a week to avoid fungal infection. The pH of nutrient solution was maintained with at 6.5 ± 0.2 throughout the experiment using H_2_SO_4_ and NaOH. Different levels of Cu were applied at juvenile stage as already previously used by Zaheer et al. (2015). In this experiment, the different levels of Cu are higher than the Cu levels applied by Habiba et al. (2015) and Azhar et al. (2006). After two weeks of giving Cu treatments, all plants were wrapped for different morphological traits, gaseous exchange attributes, antioxidants and metal accumulation in different parts of plant. All chemicals used were of analytical grade, procured from Sinopharm Chemical Reagent Co., Ltd.

### Plant harvest and sampling

After 14 days of given Cu treatment to the nutrient solution all plants were harvested for study different morphological and biochemical responses. The samples of leaves and roots were cut into small pieces for enzymatic study washed with distilled water put in liquid nitrogen immediately and stored at −80 °C for further analysis. While roots were immersed in 20 mM Na_2_EDTA for 15–20 min to remove Cu adhered to the surface of roots. Then, roots were washed thrice with distilled water and finally once with de-ionized water and dried for further analysis (Yang et al., 2004). Plant height was measured from the shoot tips to the root hairs with the help of inchi tape. Plant diameter was measured with the help of Vernier caliper (ST22302 SG Tools, Hangzhou, China). Total fresh weight was measured by measuring the weight of roots and shoots using weighting balance and plants were oven dried for 65 °C for 72 h and dry weight was measured until the weight become uniform.

### Determination of chlorophyll and gaseous exchange parameters

Total chlorophyll and carotenoid contents were determined following the method of Arnon (1949) and expressed as (mg g^−1^Fw).

On 14th day of sowing, fully expanded upper most leaves were used to measure gaseous exchange parameters i.e., P*n*, T*r*, G*s* and C*i* between 9:00 and 11:00 amusing a portable photosynthesis system Li-6400 (Li-COR, Lincoln, NE, USA).

### Determination of oxidative stress and antioxidative enzymes

The method described the concentration of lipid peroxidation was presented by Heath & Packer (1968) and expressed as nmol g^−1^ F_W_.

For the estimation of H_2_O_2_ content, “H_2_O_2_ Assay Kit” (Suzhou Comin Biotechnology Co., Ltd) was used.

The EL was measured according to the standard procedure of Dionisio-Sese & Tobita (1998).

The enzymatic activities of SOD were measured by the method of Chen & Pan (1996) and expressed in Ug^−1^ Fw.

While the enzymatic activity of POD was measured by the method of Sakharov & Ardila (1999) and expressed in Ug^−1^ Fw.

The enzymatic activity of CAT was measured by the method of Aebi (1984) and expressed in Ug^−1^ Fw.

Ascorbate peroxidase activity was measured according to Nakano & Asada (1981) and expressed in Ug^−1^ Fw.

### Determination of Cu concentration

The dried samples were grounded in to powdered form from stainless steel and 0.1 g of above dried sample was taken for digestion in HNO_3_/HClO_4_ (4:1) solution. Final readings were taken from atomic absorption spectrophotometer (AAS) model Agilent 240 FS-AA (Rehman et al., 2019c).

Bioaccumulation factor (BAF) was measured as the proportion of Cu concentration in plant tissues and Cu concentration in nutrient solution by using the following formula (Eq. (1)):

(1) \begin{eqnarray*}\mathrm{BAF}= \frac{\text{Cu concentration in plant tissues}}{\text{Cu concentration in the nutrient solution}} \end{eqnarray*}

Translocation factor (TF) was evaluate as the proportion of Cu concentration in shoots with respect to the roots as follow (Eq. (2)):

(2) \begin{eqnarray*}\mathrm{TF}= \frac{\text{Cu concentration in shoots}}{\text{Cu concentration in the roots}} \end{eqnarray*}

### Statistical analysis

All data presented in this study are the means of three replicates. The analysis of variance is set at *P* <0.05 (Kolmogorov–Smirnov test) and performed using Statistix 8.01 software and followed by Tukey’s post hoc test between the average values of treatments to find standard deviation and significant difference. Graphical representation was carried out using Sigmaplot 10 and R Studio.

## Results

### Effect of different levels of Cu on plant growth and biomass

In the present study, effect of different levels of Cu (0 (control), 60, 120 and 180 µmol L^−1^) on plant height, plant diameter, plant fresh weight and plant dry weight on *H*. *cannabinus* were also investigated (Table 1). These results suggesting that increasing levels of Cu concentrations in the nutrient solution significantly (*P* <0.05) affected plant growth and biomass of *H*. *cannabinus*. Consequently, increasing Cu levels in the nutrient solution causes a significantly reduction in plant height and plant diameter compared to the control. The maximum plant height and plant diameter reduced by 46% and 84% respectively at 180 µmol L^−1^ compared with the plants grown without Cu concentration in the nutrient solution. Similarly, plant fresh and dry biomass also affected due to high concentration of Cu in the nutrient solution and fresh and dry biomass reduced by 14% and 17% respectively at 60 µmol L^−1^, 33% and 40% respectively at 120 µmol L^−1^ and 78% and 75% respectively at 180 µmol L^−1^ compared to the control.

**Table 1:** Effect of different levels of Cu (µmol L^−1^) on plant height (cm), plant diameter (mm), plant fresh weight (g) and plant dry weight (g) on *H. cannabinus* seedlings. Values in the table is just one harvest. Mean ± SD (*n* = 3). Different letters within a column indicate significant difference between the treatments (*P* < 0.05). Relative radiance of plastic filter used: 0, 60, 120 and 180 µmol L^−1^.

Cu levels	Plant height	Plant dimeter	Plant fresh weight	Plant dry weight
0	23.6 ± 0.5a	1.6 ± 0.2a	1.6 ± 0.01a	0.7 ± 0.02a
60	19.3 ± 0.2b	1.4 ± 0.2b	1.4 ± 0.03b	0.6 ± 0.01b
120	16.1 ± 0.7c	1.2 ± 0.2c	1.2 ± 0.04c	0.5 ± 0.02c
180	12.8 ± 0.4d	1.1 ± 0.3d	0.9 ± 0.02d	0.4 ± 0.05d

### Effect of different levels of Cu on photosynthetic pigments and gaseous exchange attributes

The contents of total chlorophyll and carotenoids were significantly (*P* <0.05) affected by high concentrations of Cu in the nutrient solution (Table 2). It was noticed that maximum contents of total chlorophyll (2.9 mg g^−1^ FW) and carotenoid (0.84 mg g^−1^ FW) were observed in the treatment where the Cu concentration in the nutrient solution is 0 µmol L^−1^ while increasing Cu levels to the nutrient solution significantly reduced total chlorophyll and carotenoid contents in the leaves of *H*. *cannabinus*. Total contents of chlorophyll and carotenoid were reduced by 81% and 133% respectively were observed in the treatment of 180 µmol L^−1^ compared to the control.

**Table 2:** Effect of different levels of Cu (µmol L^−1^) on Chlorophyll a (mg g^−1^ FW), chlorophyll b (mg g^−1^ FW), total Chlorophyll (mg g^−1^ FW) and carotenoids (mg g^−1^ FW) on *H. cannabinus* seedlings. Values in the table is just one harvest. Mean ± SD (*n* = 3). Different letters within a column indicate significant difference between the treatments (*P* < 0.05). Relative radiance of plastic filter used: 0, 60, 120 and 180 µmol L^−1^.

Cu levels	Chlorophyll a	Chlorophyll b	Total Chlorophyll	Carotenoids
0	1.9 ± 0.3a	0.95 ± 0.03a	2.9 ± 0.06a	0.84 ± 0.02a
60	1.6 ± 0.3b	0.78 ± 0.02b	2.4 ± 0.05b	0.65 ± 0.02b
120	1.4 ± 0.4c	0.61 ± 0.02c	2 ± 0.06c	0.49 ± 0.01c
180	1.1 ± 0.7d	0.45 ± 0.04d	1.6 ± 0.1d	0.36 ± 0.02d

**Figure 2:**
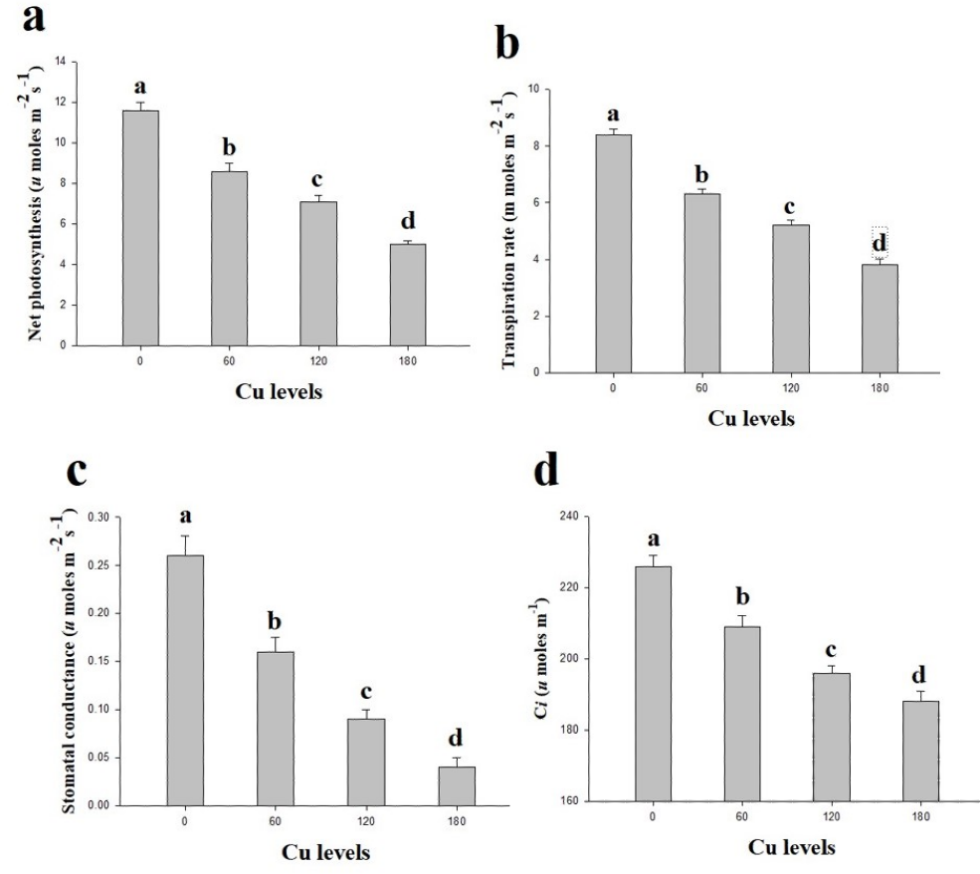
Effect of different levels of Cu on net photosynthesis (A), transpiration rate (B), stomatal conductance (C) and intercellular CO_2_ (D) on *H. cannabinus* seedlings. Values in the table are from just one harvest. Mean ± SD (*n* = 3). Different letters within a column indicate significant difference between the treatments (*P* < 0.05). Relative radiance of plastic filter used: 0, 60, 120 and 180 µmol L^−1^.

In the present study, gaseous exchange attributes were also measured from the leaves of *H*. *cannabinus* under elevating levels of Cu in the nutrient solution (Fig. 2). These results suggesting that the efficiency of P*n*, T*r*, G*s* and C*i* significantly decreases as the Cu level in the nutrient solution rises compared with the control. The maximum efficiency of P*n*, T*r*, G*s* and C*i* were observed in the plants which grown without the Cu concentration in the nutrient solution while decreased continuously as the Cu level increases in the nutrient solution. Compared to the control, P*n*, T*r*, G*s* and C*i* were reduced by 132%, 121%, 550% and 92% respectively at 180 µmol L^−1^ compared to the control.

### Effect of different levels of Cu on oxidative stress and antioxidative enzymes

**Figure 3:**
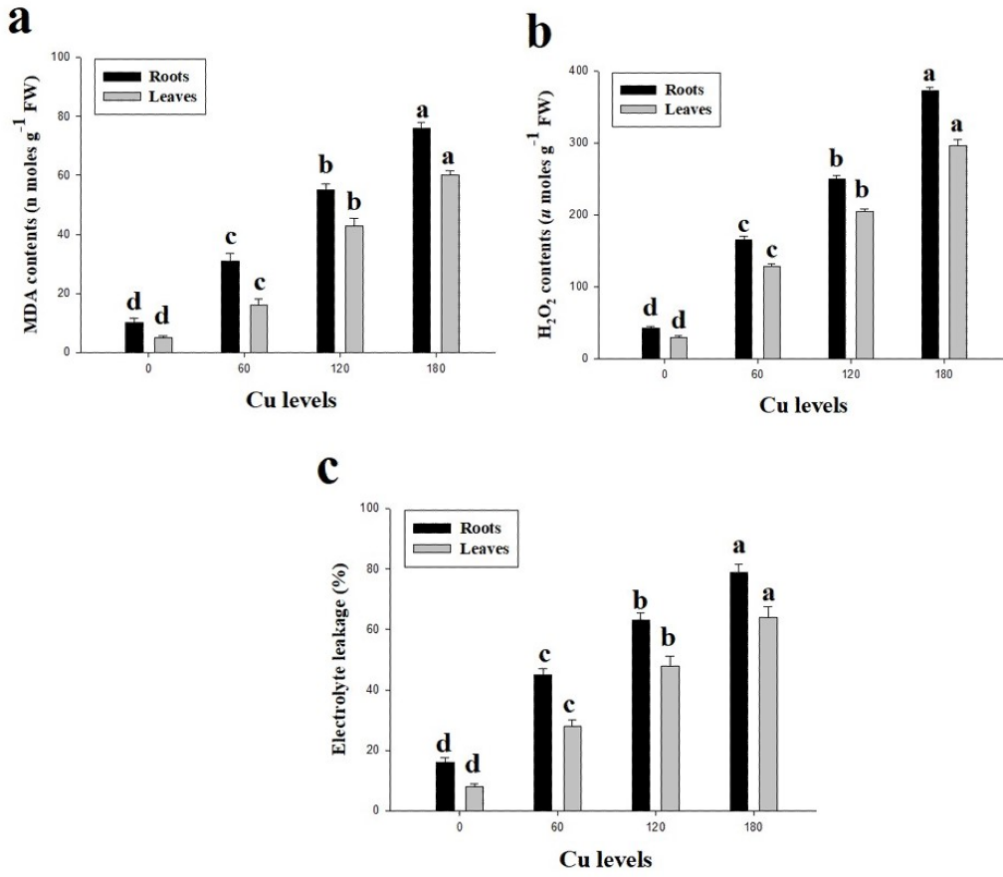
Effect of different levels of Cu on MDA (A), H_2_O_2_ (B) and EL (C) on *H. cannabinus* seedlings. Values in the table are from just one harvest. Mean ± SD (*n* = 3). Different letters within a column indicate significant difference between the treatments (*P* < 0.05). Relative radiance of plastic filter used: 0, 60, 120 and 180 µmol L^−1^.

Oxidative stress in term of high contents of malondialdehyde (MDA) and hydrogen peroxide (H_2_O_2_) were observed in this study (Fig. 3). Furthermore, percentage of electrolyte leakage (EL) were also increased in the roots and leaves of *H*. *cannabinus* compared with control. High concentration of Cu in the nutrient solution significantly (*P* <0.05) increased contents of MDA and H_2_O_2_ and EL in the roots and leaves of *H*. *cannabinus* compared with control. Compared to the control, MDA contents were increased by 660% and 1100%, H_2_O_2_ contents were increased by 767% and 886% and EL increased by 393% and 700% in the roots and leaves respectively at 180 µmol L^−1^. The minimum contents of MDA in the roots and leaves were (10 and 5 nmoles g^−1^Fw respectively), H_2_O_2_ (43 and 30 µmoles g^−1^Fw respectively) and EL (16 and 8% respectively).

**Figure 4:**
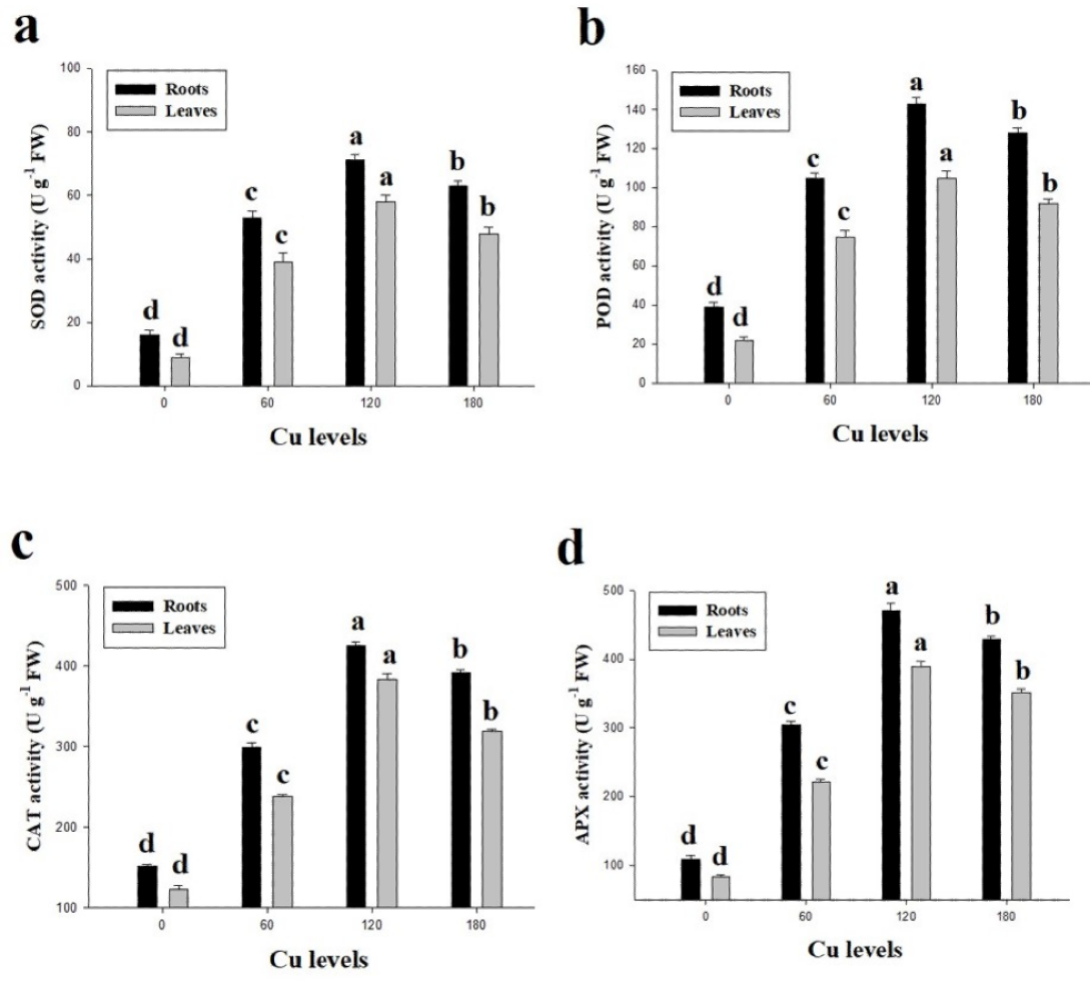
Effect of different levels of Cu on SOD (A), POD (B), CAT (C) and APX (D) on *H. cannabinus* seedlings. Values in the table are from just one harvest. Mean ± SD (*n* = 3). Different letters within a column indicate significant difference between the treatments (*P* < 0.05). Relative radiance of plastic filter used: 0, 60, 120 and 180 µmol L^−1^.

In the present study, antioxidative enzymes (SOD, POD, CAT and APX) from the roots and leaves of *H*. *cannabinus* under elevating levels of Cu (0 (control), 60, 120 and 180 µmol L^−1^) were also investigated (Fig. 4). It was noticed that the antioxidative enzymes were initially increased when plants subjected to the Cu levels of 60 and 120 µmol L^−1^while further increase in Cu concentration in the nutrient solution causes a significantly reduction in the antioxidants measured from roots and leaves of *H*. *cannabinus*. Our results depicted the antioxidants i.e., SOD, POD, CAT and APX increased in the roots by 343%, 228%, 159% and 294% respectively while in the leaves increased by 433%, 318%, 160% and 366% respectively in the leaves compared to the control. Compared with the control, the maximum activity of SOD, POD, CAT and APX were observed in the plant grown under Cu level of 120 µmol L^−1^ followed by 180 µmol L^−1^ and 60 µmol L^−1^.

### Cu uptake from different parts of plant

In the present study, Cu concentration from different parts (roots, leaves and stems) of *H*. *cannabinus* under elevating levels of Cu (0 (control), 60, 120 and 180 µmol L^−1^) in the nutrient solution were also determined (Table 3). These results depicted that increasing Cu concentration in the nutrient solution enhances Cu concentration in roots, leaves and stems of *H*. *cannabinus*. Furthermore, these were also suggesting that the maximum Cu was accumulated in the roots while a little transported to the harvestable parts (leaves and stems) of the plants (Table 3). Compared to the control, the maximum increased in Cu concentration was observed in the roots (103 mg/kg) followed by leaves (88 mg/kg) and stems (75 mg/kg). *H*. *cannabinus* can accumulate Cu in the range of 6–103, 4–88 and 5–75 mg/kg in the roots, leaves and stems respectively.

**Table 3:** Cu accumulation in roots (mg/kg), leaves (mg/kg) and stems (mg/kg) on *H. cannabinus* seedlings grown under different levels of Cu (µmol L^−1^). Values in the table is just one harvest. Mean ± SD (*n* = 3). Different letters within a column indicate significant difference between the treatments (*P* < 0.05). Relative radiance of plastic filter used: 0, 60, 120 and 180 µmol L^−1^.

Cu levels	Roots	Leaves	Stems
0	6 ± 1.4d	4 ± 1.4d	3 ± 1.4d
60	33 ± 2.5c	25 ± 2.5c	18 ± 1.4c
120	68 ± 2.5b	58 ± 1.8b	45 ± 2.5b
180	103 ± 6a	88 ± 1.8a	75 ± 2.5a

**Figure 5:**
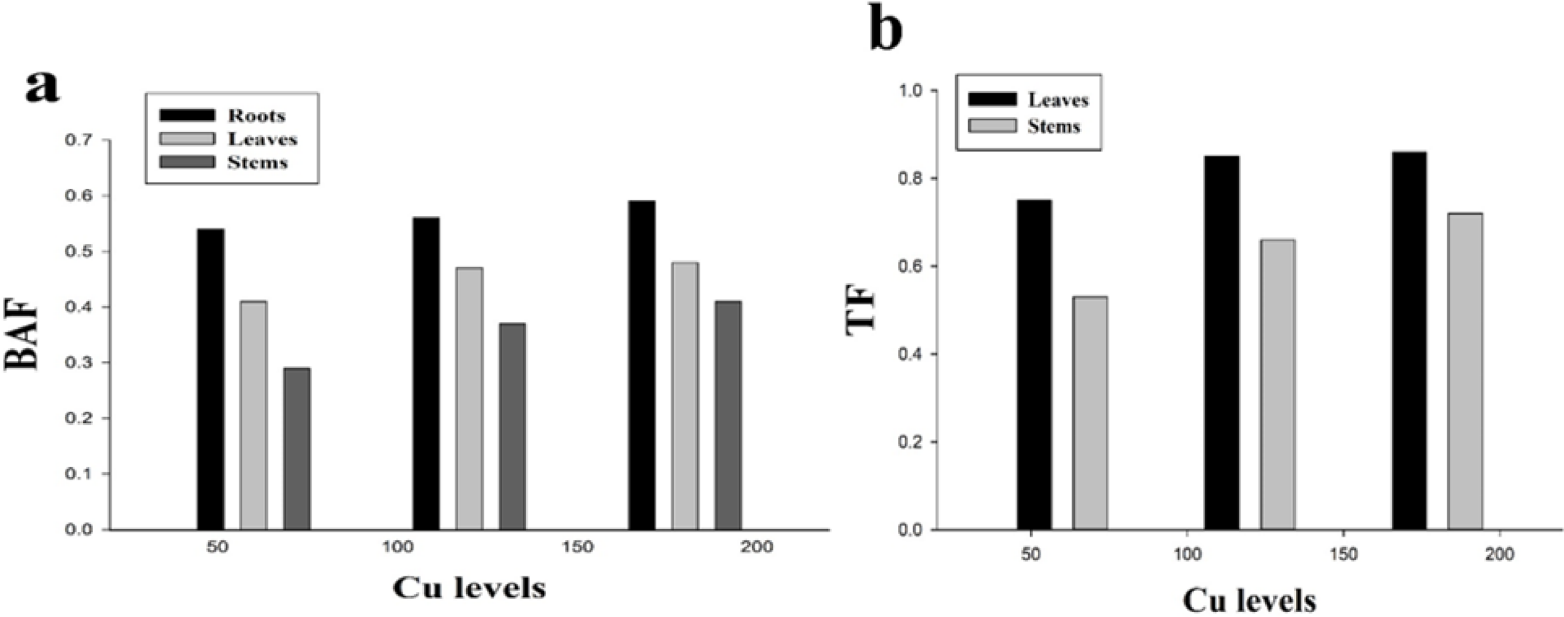
Effect of different levels of Cu on BAF (A) and TF (B) on *H. cannabinus* seedlings. Values in the table are from just one harvest. Mean ± SD (*n* = 3). Different letters within a column indicate significant difference between the treatments (*P* < 0). Relative radiance of plastic filter used: 0, 60, 120 and 180 µmol L^−1^.

BAF and TF could be used to assess the level phytoextraction of a plant. The results regarding BAF and TF are presented in (Fig. 5). It was noticed that all the values of BAF and TF were less than 1 while minimum values of TF in the leaves (0.86) and stems (0.72) were recorded at 180 µmol L^−1^. The highest values of BAF were recorded at 180 µmol L^−1^ which were 0.59, 0.48 and 0.41 in the roots, leaves and stems respectively while the minimum values of BAF were recorded at 60 µmol L^−1^ which were 0.54, 0.41 and 0.29 in the roots, leaves and stems respectively. *H*. *cannabinus* accumulate highest Cu concentration in the roots followed by leaves and stems.

### Relationship between growth parameters and Cu uptake

**Figure 6:**
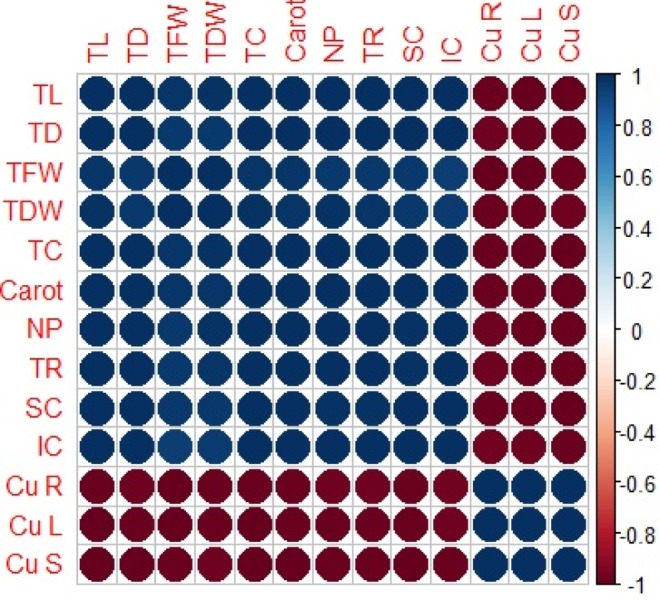
Relationship between growth parameters and photosynthetic pigments, gaseous exchange attributes and Cu uptake from different parts of *H. cannabinus*. TL (total length), TD (total diameter), TFW (total fresh weight), TDW (total dry weight) TC (total c).

A Pearson correlation analysis was carried out to quantify the relationship between growth parameters, photosynthetic pigments, gaseous exchange attributes and Cu uptake from different parts (roots, leaves and stems) of *H*. *cannabinus* (Fig. 6). Cu concentration in the roots is positively correlated with Cu concentration in the leaves while negatively correlated with plant height, plant diameter, plant fresh weight, plant dry weight, total chlorophyll, carotenoid contents, net photosynthesis, transpiration rate, stomatal conductance and intercellular CO_2_. Similarly, Cu concentration in the leaves is positively correlated with cu concentration in the roots and stems while negatively correlated with all other parameters of the plant. This correlation reflected the close connection between Cu uptake and growth in *H*. *cannabinus* seedlings.

## Discussion

Phytotoxicity of copper (Cu) can cause poor seed germination, stunned plant height, reduce root growth and ultimately affected plant yield and crop productivity (Celis-Plá et al., 2018; Gao et al., 2017; Husak, 2015; Rehman et al., 2019c). A lot of investigation already done on different plant species that high concentration of Cu in the medium affect the plant growth and biomass (Chen et al., 2015b; Husak, 2015; Ibrahim, Chee Kong & Mohd Zain, 2017; Liu et al., 2018). In the present study, it was observed that excess Cu in the nutrient solution reduced plant growth and biomass compared to the control (Table 1). Phytotoxicity of Cu on plant growth and biomass of many plant species has been reviewed by Adrees et al. (2015a) and Adrees et al. (2015b). In a previous study, under the application of 100 µmol L^−1^, *Zea mays* reduced roots, shoot and leaf length due to high concentration of Cu in the nutrient solution (Benimeli et al., 2010). In another study, the application of 15 µmol L^−1^ reduced root length by 90% while application of 157 µmol L^−1^ almost died the of 14 days old plant (Barbosa et al., 2013). Furthermore, application of 50 µmol L^−1^ to 6-day old plant of reduced shoot length by 31% compared with control (Gang, Vyas & Vyas, 2013). However, the degree of damage of growth and biomass of *H*. *cannabinus* is relatively low which indicated that this plant can tolerate under toxic levels of Cu in the soil (Table 1). The decrease in plant growth and biomass under toxic levels of Cu in the nutrient solution might be due to decrease in nutrient availability from the nutrient solution and increase in Cu concentration in the roots, leaves and stems of *H*. *cannabinus*.

Photosynthetic pigments of a plant are important biological parameters in elevating of abiotic stress (Rehman et al., 2019c; Wodala et al., 2012). In the present study, photosynthetic pigments and gaseous exchange attributes affected due to high concentration of Cu in the nutrient solution (Table 2 and Fig. 2). The decrease in photosynthesis might be due to the inhibition activities of some enzymes associated with chlorophyll biosynthesis (Brahim & Mohamed, 2011; Chen et al., 2015b; Ibrahim, Chee Kong & Mohd Zain, 2017). One more possible reason behind this mechanism is displacement of Mg which required for chlorophyll biosynthesis (Zaheer et al., 2015) which causes alteration in ultra-structure of chloroplast (Azhar et al., 2006; Habiba et al., 2015). Furthermore, phytotoxicity of Cu causes damage to photosynthetic machinery could be associated with translocation of Cu in to the leaves which could impair lamellar membrane of chloroplast (Najeeb et al., 2009). These findings are coinciding with the finding of (Zaheer et al., 2015) who noticed that elevating level of Cu (50 and 100 µmol L^−1^) in the nutrient solution significantly reduced chlorophyll contents and rate of photosynthesis in *Brassica napus*.

Excess Cu causes free Cu ions in the cell/tissues of the plants which are involved in the generation of reactive oxygen species (ROS) such as singlet oxygen (^1^O_2_) and hydroxyl radicals (OH) (Ibrahim, Chee Kong & Mohd Zain, 2017; Kohli et al., 2018; Kosar, Akram & Ashraf, 2015; Rehman et al., 2019a). These ROS can cause progressive oxidative damage and ultimately cell death. The enzymatic activities of antioxidants such as SOD, POD, CAT and APX are involved in the scavenging of ROS. For example, SOD catalyzes ^1^O_2_ to H_2_O_2_ and POD decomposes H_2_O_2_ to H_2_O (Habiba et al., 2015; Li et al., 2013; Singh et al., 2017). Furthermore, high concentration of Cu in the medium causes oxidative damage and could support ion leakage by plasmalemma membrane (Kamran et al., 2019; Zaheer et al., 2015). The results from the present study is revealed that increase in lipid peroxidation and oxidative stress in the leaves of *H*. *cannabinus* (Fig. 3). The increase in lipid peroxidation, H_2_O_2_ and EL in the roots and leaves of *H*. *cannabinus* might be due to the progressive increase of Cu levels in the nutrient solution which is a stress factor triggering physiological responses in plants (Chen et al., 2015b; Rehman et al., 2019b). The up-regulation activities of various antioxidants (SOD, POD, CAT and APX) (Fig. 4) shows the capacity of plant to scavenge ROS (Rehman et al., 2019a; Zaheer et al., 2015). Increased the activities of antioxidants in the roots and leaves of *H*. *cannabinus* under low level of Cu (60 and 120 µmol L^−1^) in the nutrient solution indicating that *H*. *cannabinus* could tolerate stress levels to a certain level by enhancing antioxidative defense system. The variable response of antioxidants on the roots and leaves of *H*. *cannabinus* might be due to the alteration in gene expression and function of various proteins associated with different physiological structure in plants (Chen et al., 2015a; Gao et al., 2017; Kohli et al., 2018). However, decreased in the activities of antioxidants under higher levels of Cu might be due to severe oxidative stress as previously reported by Habiba et al. (2015).

The uptake and translocation of Cu to different parts of plant (roots, leaves and stems) is usually depend upon growth condition, metal supply and plant species in the nutrient solution (Husak, 2015; Rehman et al., 2019b). Cu cause toxicity usually in roots and its morphology because Cu is highly accumulated in the belowground parts (roots) while a little transported to the aboveground parts (leaves, stems, and seeds) of the plant. It was also reported that translocation of Cu to harvestable parts of the plants is usually restricted due to its highly accumulation in the belowground parts of the plant (Husak, 2015; Rehman et al., 2019a). The results from the present study is suggesting that maximum Cu concentration was accumulated in the roots (103 mg/kg) followed by leaves (88 mg/kg) and stems (75 mg/kg) at 180 µmol L^−1^ (Table 3). These findings are greater than the normal concentration of Cu in the soil i.e., 20–30 mg/kg as showed by Rehman et al. (2019c) which indicating that *H*. *cannabinus* can accumulate high concentration of Cu in their body parts when grown under Cu contaminated soil. However, metal uptake in different parts of plant also depend upon xylem tissue loading, detoxification and transport across cell membrane at cellular level. The present result similar to the results of Sultana & Kobayashi (2011), who reported that under metal contaminated environment metal tend to accumulate higher in the roots of plants while a little transport to the above ground parts. Li et al. (2013) studied *H*. *cannabinus* seedlings under Cd stress in the nutrient solution and observed that short-term application of Cd is highly accumulated in the roots while a little transport to the shoots. BAF and TF are the most biological parameters in screening the phytoremediation potential of a plant. The plant might be a good hyperaccumulator specie when both values of BAF and TF should be greater than 1 (Chen et al., 2015b; Muszynska & Hanus-Fajerska, 2015; Yang et al., 2004). Contrastingly, Uddin et al. (2016) studied different varieties of *H*. *cannabinus* plant under Pb contaminated soil and resulted that Pb was highly accumulated in the harvestable parts of the plant while little accumulated in belowground parts. However, Santos et al. (2010) studied *H*. *cannabinus* plant under different heavy metals (Zn, Cu, Mn, Pb and B) and resulted that heavy metals were accumulated by *H*. *cannabinus* plants in following order Zn >B >Mn >Pb >Cu in the shoots and roots in dry biomass of the plant. Our results revealed that the values of BAF and TF are less than 1 this is because of Cu was highly accumulated in the roots while a little transported to the leaves and stem (Fig. 5). The possible reason behind it is *H*. *cannabinus* undergoes Cu stress for short period (14 days) while Bhattacharya et al. (2014) noticed that short term metal stress causes metal accumulation in the roots while long term metal stress support plant to able to accumulate in the above ground parts. Although, BAF and TF cannot evaluate alone the phytoremediation of the plant, biomass of the plant is also important in screening the phytoremediation potential against stress tolerance (Chen et al., 2015a; Chen et al., 2015b). Similar results showed by Zaheer et al. (2015) that short-term heavy metal application was highly accumulated in the roots while a little transported to the shoots.

## Conclusion

Based on these findings, it can be concluded that *H*. *cannabinus* can cope Cu stress due to active antioxidant defense system. However, elevating concentration of Cu (60, 120 and 180 µmol L^−1^) in the nutrient solution causes significant reduction in plant height, plant diameter, plant fresh weight, plant dry weight, total chlorophyll, carotenoid contents and gaseous exchange attributes. Furthermore, phytotoxicity of Cu in the nutrient solution causes lipid peroxidation by enhancing contents of MDA which induced oxidative damage in *H*. *cannabinus* seedlings. The antioxidants (SOD, POD, CAT and APX) come into play to scavenge ROS by enhancing their activities up to 120 µmol L^−1^ while a further increase in Cu concentration (180 µmol L^−1^) causes a decrease in antioxidant activities. Moreover, elevating levels of Cu also increases Cu concentration in roots, leaves and stems of *H*. *cannabinus* while cu was highly accumulated in the roots followed by leaves and stems. Only 14 days old seedlings of *H*. *cannabinus* were able to accumulate high concentration of Cu in their body parts, so we suggest that *H*. *cannabinus* has a great potential to accumulate Cu in their body parts and can be used as a phytoremediation tool under Cu contaminated soil.

##  Supplemental Information

10.7717/peerj.8321/supp-1 Data S1 Raw Data

## Additional Information and Declarations

### Data Availability

The following information was supplied regarding data availability: The raw data are in available in the Supplemental File.
